# 3,4-Dihydroxyphenylglycol levels separate multiple system atrophy from Parkinson disease with orthostatic hypotension

**DOI:** 10.1007/s10286-025-01150-8

**Published:** 2025-09-17

**Authors:** David S. Goldstein, Patti Sullivan, Courtney Holmes

**Affiliations:** Autonomic Medicine Section, Clinical Neurosciences Program, Division of Intramural Research, National Institute of Neurological Disorders and Stroke, National Institutes of Health, 10 Center Drive MSC-1620, Building 10 Room 8N260, Bethesda, MD 20892-1620 USA

**Keywords:** DHPG, Parkinson, Multiple system atrophy, Orthostatic hypotension, Sympathetic

## Abstract

**Background:**

The autonomic synucleinopathy multiple system atrophy (MSA) can be difficult to distinguish clinically from Parkinson disease with orthostatic hypotension (PD+OH). ^18^F-Dopamine positron emission tomography separates these conditions based on cardiac noradrenergic deficiency in PD+OH and not in MSA but is available only at the NIH Clinical Center. 3,4-Dihydroxyphenylglycol (DHPG) is the main neuronal metabolite of norepinephrine. This retrospective observational study examined whether DHPG levels in cerebrospinal fluid (CSF) or plasma differentiate MSA from PD+OH.

**Methods:**

We reviewed CSF and plasma neurochemical data from all patients referred for evaluation at the NIH Clinical Center between 1995 and 2024 for chronic autonomic failure or parkinsonism. A concurrently studied comparison group included healthy volunteers or patients with orthostatic intolerance.

**Results:**

CSF DHPG was decreased in MSA (*N* = 67, *p* < 0.0001) compared to the controls but also tended to be decreased in PD+OH (*N* = 31, *p* = 0.0776). Antecubital venous plasma DHPG was decreased in PD+OH (*N* = 47, *p* = 0.0064) but not in MSA. CSF/plasma concentration ratios of DHPG were lower in MSA than in PD+OH (*p* = 0.0005). Cardiac arteriovenous increments in plasma DHPG and cardiac norepinephrine spillovers were strikingly decreased in PD+OH (*N* = 6) and were lower than in MSA (*N* = 20, *p* < 0.0001 each). Combining cardiac arteriovenous increments in plasma DHPG with norepinephrine spillovers completely separated PD+OH from MSA.

**Conclusions:**

CSF/plasma ratios of DHPG, cardiac arteriovenous increments in plasma DHPG, and cardiac norepinephrine spillovers separate MSA from PD+OH. On the basis of our results we propose that biomarker combinations involving DHPG in biofluids may enable a clinical laboratory distinction of MSA from PD+OH.

## Introduction

Multiple system atrophy (MSA) and Parkinson disease (PD) are synucleinopathies involving intra-cytoplasmic deposition of the protein alpha-synuclein (α-syn). In MSA the deposits are in glial cytoplasmic inclusions [1], whereas in PD α-syn is deposited in Lewy bodies [2]. MSA and PD often entail clinical manifestations of chronic autonomic failure [3] and are classified as autonomic synucleinopathies. Neurogenic orthostatic hypotension (nOH) occurs in a substantial minority of PD [4] and in most MSA [5] patients.

It is difficult to distinguish PD with OH (PD+OH) from MSA by clinical criteria alone, especially early in the disease course. There is an ongoing need for pathophysiologically relevant biomarkers that can differentiate the two conditions. ^18^F-Dopamine (^18^F-DA) positron emission tomography (PET) to identify cardiac noradrenergic deficiency efficiently separates MSA from PD+OH [6], but for many years this testing modality has been available only at the NIH Clinical Center. Moreover, ^18^F-DA PET is investigational, expensive, and involves radioactivity exposure. Several recent studies have reported on CSF α-syn seeding activity [7, 8] or α-syn deposition in skin biopsies [9–11], but so far it is unclear how well these tests separate MSA and PD+OH from each other and from other autonomic disorders.

Both MSA and PD+OH feature central noradrenergic deficiency, as indicated in vivo by decreased cerebrospinal fluid (CSF) levels of norepinephrine (NE) and its metabolites [12] and post-mortem by decreased putamen tissue NE contents [13]. In the periphery, orthostatic increments in plasma NE are attenuated in both MSA [14] and PD+OH [15], reflecting baroreflex-sympathoneural failure.

MSA and PD+OH differ in the occurrence of sympathetic noradrenergic deficiency [16], especially in the heart. In vivo and post-mortem data have consistently indicated a substantial cardiac sympathetic noradrenergic lesion in PD+OH and intact sympathetic innervation in most (but not all) patients with MSA [17, 18]. The difference in peripheral abnormalities despite similar central noradrenergic abnormalities provided the theoretical backdrop for the present study.

3,4-Dihydroxyphenylglycol (DHPG) is the main neuronal metabolite of NE, and plasma DHPG in humans is derived mainly from sympathetic nerves. Under resting conditions the main determinant of plasma DHPG is net leakage of NE from storage vesicles into the axonal cytoplasm, a process quite different from exocytotic release of NE due to post-ganglionic sympathetic nerve traffic [19]. Simultaneous measurements of plasma NE and DHPG provide complementary information about the functional status of sympathetic noradrenergic nerves [20]. Analogously, DHPG in CSF provides a neurochemical “window” on the synthesis, storage, and metabolism of NE in the central nervous system [21].

Based on the above considerations, in this retrospective observational study we hypothesized that CSF/plasma ratios of NE and DHPG, cardiac NE spillovers, and cardiac venous–arterial differences in plasma DHPG would distinguish PD+OH from MSA.

## Methods

### Study subjects

All the participants in this study gave written informed consent before any research procedures were done. The protocols were approved by the Institutional Review Board of the National Institute of Neurological Disorders and Stroke (NINDS) or of the National Institutes of Health (NIH). All the patients had been referred to the Autonomic Medicine Section (formerly the Clinical Neurocardiology Section) of the Division of Intramural Research of the NINDS for known or suspected autonomic dysfunction and were studied at the NIH Clinical Center. Accrual was by referral only; there was no recruitment by advertisement.

The presence or absence of neurogenic OH (nOH) was determined based on beat-to-beat blood pressure responses to the Valsalva maneuver or orthostatic fractional increments in plasma NE levels [22, 23].

Patients were stratified into PD+OH or MSA groups based on previously published consensus statements [24–27]. We also used the UK Brain Bank criteria for PD [28], with an important exception. According to the UK Brain Bank criteria, early, prominent autonomic involvement is exclusionary for diagnosing PD. Findings by our group [29–31] and others [32] that OH can be an early finding in PD disagree with this assertion. Most of the patients underwent cardiac sympathetic neuroimaging by ^18^F-DA PET, which efficiently separates PD+OH from MSA, in that virtually all patients with PD+OH have low myocardial ^18^F- DA-derived radioactivity, whereas most patients with MSA have normal radioactivity [6, 18].

For comparison with the PD+OH and MSA groups data were culled from healthy volunteers and from patients referred for chronic orthostatic intolerance who did not have evidence of chronic autonomic failure. The healthy volunteers were recruited through the NIH Normal Volunteer Program or were self-referred. All the control subjects had unremarkable results of routine screening labs, and none had diabetes mellitus.

### Blood sampling

With the subject supine an intravenous catheter was placed percutaneously in an arm vein, usually an antecubital vein. A three-way stopcock was attached to the hub of the catheter. Normal saline was infused at a slow rate to keep the vein open. After the subject had been supine for at least 15 min, about 1 mL of blood was drawn through the stopcock and discarded. About 5 mL of blood was then sampled and placed in ice. The blood was centrifuged in a refrigerated centrifuge, and the plasma was transferred to a plastic cryotube and stored at −80 °C until thawed for assay.

### Lumbar puncture

Lumbar puncture was performed under fluoroscopic guidance by a board-certified neuroradiologist or by a neuroradiology post-doctoral fellow. Aliquots of 1 mL of CSF were frozen immediately in dry ice and kept frozen at −80 °C until thawed for assay. In most cases the sixth aliquot was assayed for catechols.

### Right heart cardiac catheterization

Right heart catheterization was performed by a team led by a board-certified cardiologist who was credentialed at the NIH Clinical Center. A left brachial arterial catheter was placed for monitoring blood pressure and obtaining blood samples. The right internal jugular vein was cannulated percutaneously after anesthesia of the overlying skin, and a plastic catheter was advanced into the great cardiac vein or coronary sinus. The configuration of the catheter was the reverse of a Swan–Ganz catheter; saline injectate at room temperature was administered distal rather proximal to the thermistor, enabling measurement of coronary sinus blood flow by thermodilution. Tracer-labeled NE was infused continuously into a systemic vein. After at least 20 min of supine rest, an arterial and a cardiac venous blood sample were obtained approximately simultaneously.

The term “arteriovenous increment” refers to the increase in the concentration of an analyte between the arterial plasma and the local venous plasma. “Spillover” refers to the calculated rate of entry into the venous drainage of the heart, in units of pmol/min.

The present study relied on cardiac catheterization data from a previously published study [33]. Relationships among arm and cardiac arteriovenous increments in plasma DHPG and cardiac NE spillover in MSA vs. PD+OH have not been reported previously.

### ^18^F-Dopamine PET

Most of the patients underwent cardiac sympathetic neuroimaging by ^18^F-DA PET as reported previously [33]. Briefly, 1 mCi of ^18^F-DA was infused intravenously over 3 min. Interventricular septal myocardial ^18^F-DA-derived radioactivity was measured in the 5′ dynamic frame with the midpoint about 8′ after initiation of administration of the tracer. Radioactivity concentrations in nCi/cc were adjusted for the dose injected per kg, so that the radioactivity concentrations were in units of nCi-kg/cc-mCi. Previous studies have not examined relationships of CSF/plasma ratios of DHPG levels to cardiac ^18^F-DA-derived radioactivity.

### Catechol assay

Plasma and CSF were assayed by batch alumina extraction followed by liquid chromatography with series electrochemical detection as described previously by our group [34, 35].

### Avoidance of biases

Personnel conducting neurochemical assays or analyses of PET images were blinded as to the other clinical laboratory results until the data were tabulated.

### Data analysis and statistics

Cardiac NE spillover (in pmol/min) was calculated as described previously [36] using the tracer dilution principle. Briefly, a tracer amount of ^3^H-NE was infused to a steady state (≥ 20 min), and cardiac NE spillover was calculated according to the equation

$${\text{NE Spillover }} = {\text{Q}} \,{*} \left( {{\text{NEv }}{-}{\text{ NEa}}}\right) \,{*} \,({\text{SAa}}{/} {\text{SAv}}),$$

where Q = coronary sinus plasma flow (in mL/min), NEv = cardiac venous NE concentration (in pmol/mL), NEa = arterial NE concentration (in pmol/mL), SAa = specific activity of ^3^H-NE in arterial plasma, and SAv = specific activity of ^3^H-NE in cardiac venous plasma.

For statistical analyses and graphics GraphPad Prism 9 for Mac (GraphPad Software, Boston, MA) was used.

Mean values in the PD+OH, MSA, and control groups were compared by factorial analyses of variance with Tukey’s post hoc test. Pearson correlation coefficients were calculated for scatterplots. Receiver operating characteristic (ROC) curves were generated for evaluating the efficiency of CSF/plasma ratios of DHPG in separating MSA from PD+OH and from controls. Frequencies were analyzed by Fisher’s exact test. A *p* value less than 0.05 defined statistical significance.

## Results

Data were reviewed from a total of 241 participants (87 MSA, 47 PD+OH, 40 controls with chronic orthostatic intolerance, 67 healthy volunteers). Demographic data for the groups are in Table 1. All the patients were off levodopa/carbidopa as documented by CSF or plasma DOPA levels within the respective normal ranges.

**Table 1:** Group demographics

Group	Age quartile	Years	Number	Male/female
Control (COI)	Quartile 4	58	6	Male
	Quartile 3	45	40	Female
	Quartile 2	37	85%	% Female
	Quartile 1	28		
Control (HV)	Quartile 4	85	45	Male
	Quartile 3	57	67	Female
	Quartile 2	46	33%	% Female
	Quartile 1	33		
MSA	Quartile 4	79	56	Male
	Quartile 3	66	87	Female
	Quartile 2	60	36%	% Female
	Quartile 1	54		
PD+OH	Quartile 4	81	33	Male
	Quartile 3	74	47	Female
	Quartile 2	69	30%	% Female
	Quartile 1	63		

Quartile 4 = maximum value; Quartile 2 = median value; Quartile 1 = minimum value

*COI* chronic orthostatic intolerance, *HV* healthy volunteer, *MSA* multiple system atrophy, *PD*+*OH* Parkinson disease with orthostatic hypotension

The vast majority of MSA patients had orthostatic hypotension. Of 81 MSA patients with relevant historical information available, 66 (81%) reported a history of orthostatic hypotension, and of 58 MSA patients who underwent head-up tilt table testing for 5 min, 50 (86%) had an orthostatic fall in systolic blood pressure of at least 20 mm Hg. Of 49 MSA patients who had blood pressure recorded continuously during and after performing the Valsalva maneuver, 47 (96%) had a progressive fall in systolic pressure during Phase II and a lack of overshoot in pressure in Phase IV, documenting baroreflex-sympathoneural dysfunction. These cardiovascular autonomic abnormalities overlapped almost completely with those found in PD+OH.

**Fig. 1:**
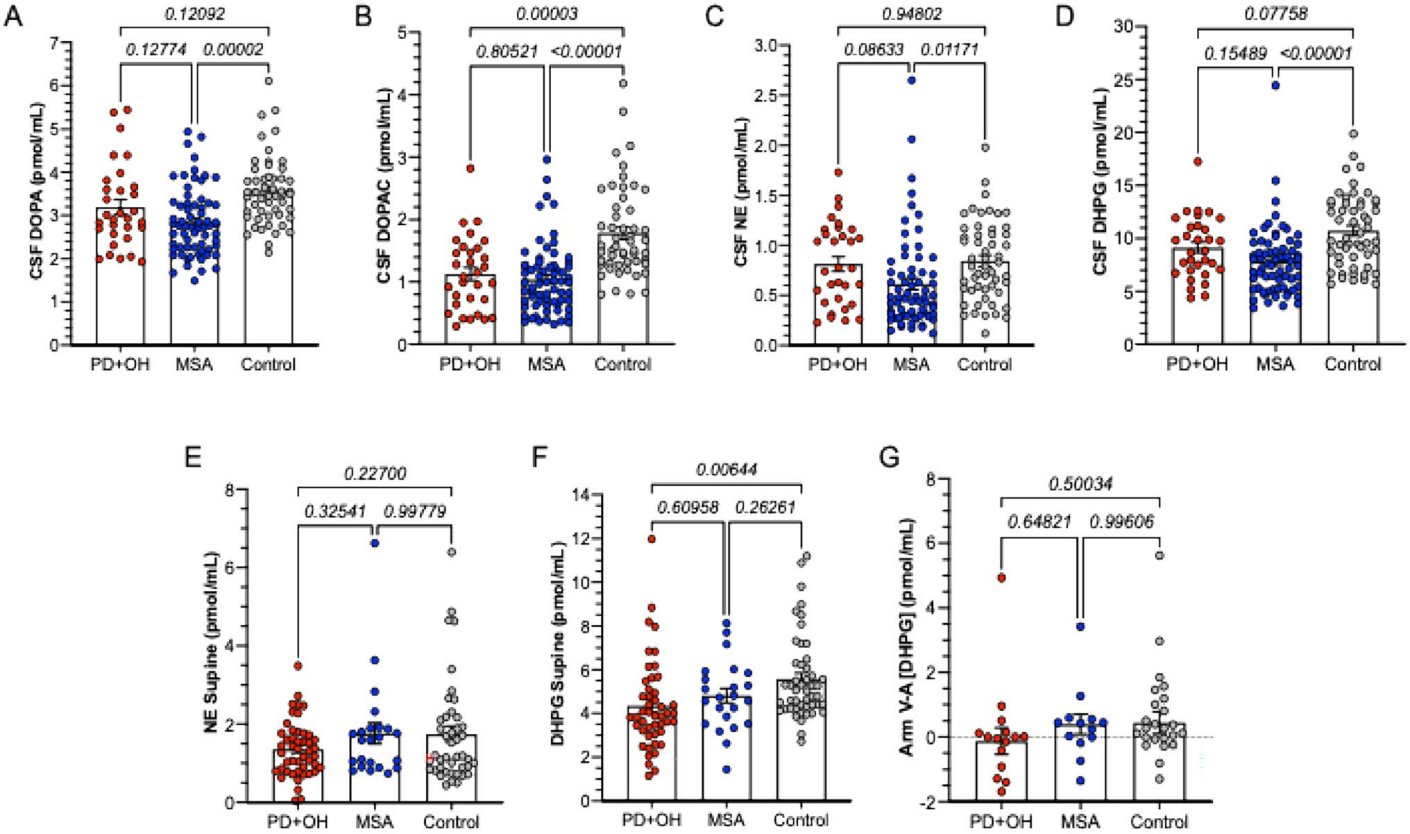
Individual and mean (± SEM) values for cerebrospinal fluid (CSF) and plasma catechols in patients with Parkinson disease and orthostatic hypotension (PD+OH, red), multiple system atrophy (MSA, blue), and control subjects (Control, gray). **A** CSF DOPA; **B** CSF 3,4-dihydroxyphenylacetic acid (DOPAC); **C** CSF norepinephrine (NE); **D** CSF 3,4-dihydroxyphenylglycol (DHPG); **E** plasma NE during supine rest; **F** plasma DHPG during supine rest; **G** Arm venous–arterial (V–A) difference in plasma DHPG. Numbers in italics are *p* values for group comparisons by Tukey’s test. In MSA, but not in PD+OH, CSF DOPA was decreased compared to controls. CSF DOPAC was decreased in both MSA and PD+OH. CSF NE was decreased in MSA but not in PD+OH. CSF DHPG was decreased in MSA and tended to be decreased in PD+OH. Plasma NE was not decreased in PD+OH or MSA. Plasma DHPG was decreased in PD+OH but not in MSA. The groups did not differ in venous–arterial differences in arm DHPG

Compared to values in the control group, in the MSA group CSF DOPA, DOPAC, NE, and DHPG were decreased (Fig. 1A–1D), while in the PD+OH group CSF DOPAC was decreased but not CSF DOPA, NE, or DHPG. Antecubital venous plasma NE was not decreased in PD+OH or MSA (Fig. 1E). Plasma DHPG was decreased in the PD+OH but not in the MSA group (Fig. 1F). The groups did not differ in arm arteriovenous increments in plasma DHPG levels (Fig. 1G).

**Fig. 2:**
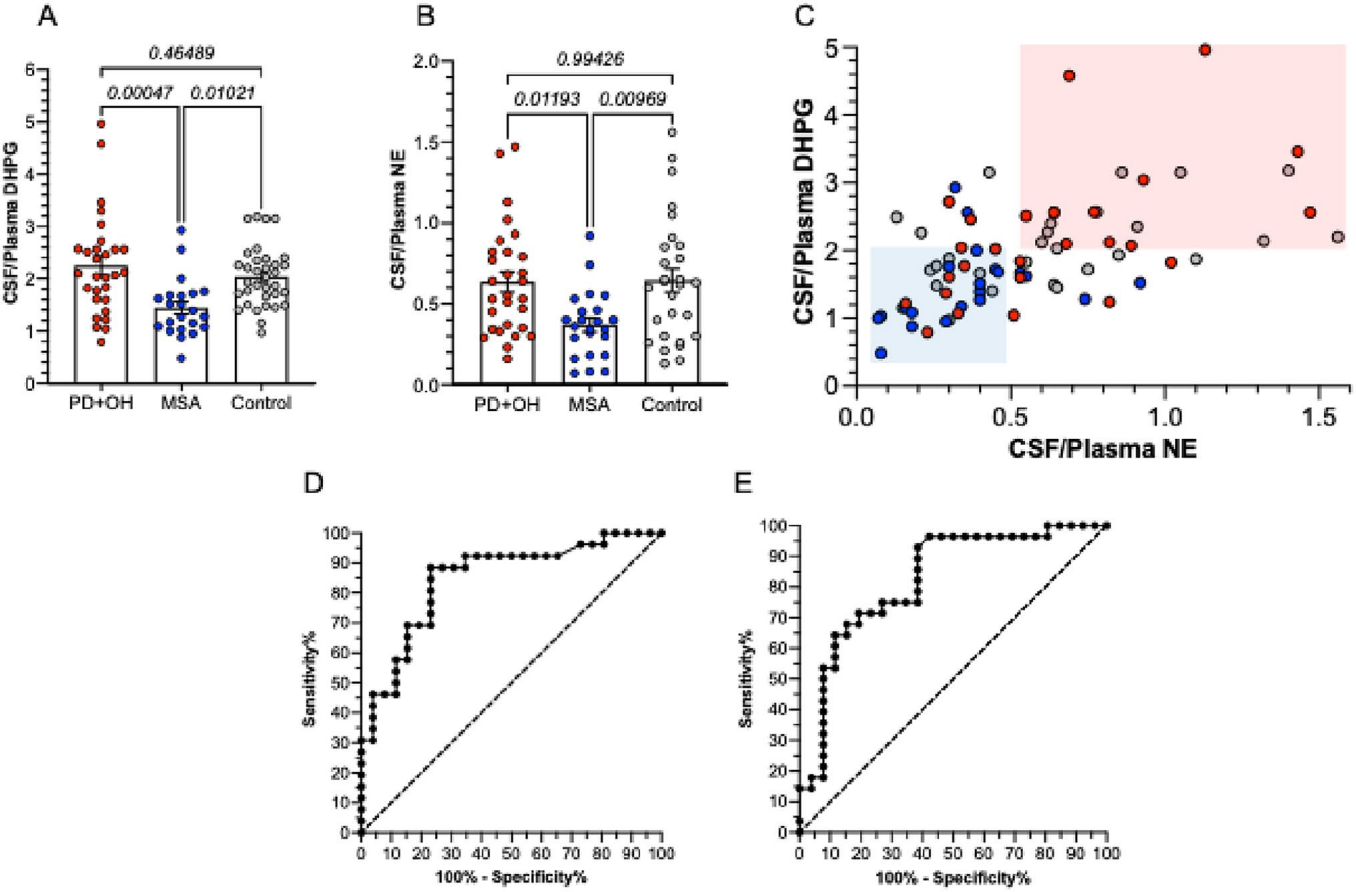
CSF/plasma ratios in multiple system atrophy (MSA, blue), Parkinson disease with orthostatic hypotension (PD+OH, red), and control subjects (gray). **A** CSF/plasma ratios of 3,4-dihydroxyphenylglycol (DHPG); **B** CSF/plasma ratios of norepinephrine (NE); **C** scatterplot relating individual data for CSF/plasma ratios of DHPG to data for CSF/plasma ratios of NE; **D** Receiver Operating Characteristic (ROC) curves for CSF/plasma ratios of DHPG in MSA vs. PD+OH; **E** ROC curves for CSF/plasma ratios of DHPG in MSA vs. controls. In **C**, blue and pink rectangles are placed manually to highlight the separation of MSA from PD+OH (*p* < 0.0001 by Fisher’s exact test). In **A**, data were excluded from one MSA patient with a high CSF/plasma ratio of DHPG. In **B**, data were excluded from two PD+OH patients with high CSF/plasma ratios of NE

CSF/plasma ratios of both DHPG (Fig. 2A) and NE (Fig. 2B) were lower in MSA than in PD+OH, with the group difference most noticeable for CSF/plasma ratios of DHPG. CSF/plasma ratios of DHPG and NE considered together separated MSA patients from PD+OH patients (blue and pink rectangles in Fig. 2C. ROC analyses for 26 MSA vs. 26 PD+OH patients showed a ROC area of 0.8476 (95% confidence interval 0.7409–0.9544, *p* < 0.0001; Fig. 2D). For 26 MSA patients vs. 28 controls the ROC area was 0.8249 (95% confidence interval 0.7111–0.9387, *p* < 0.0001; Fig. 2E).

**Fig. 3:**
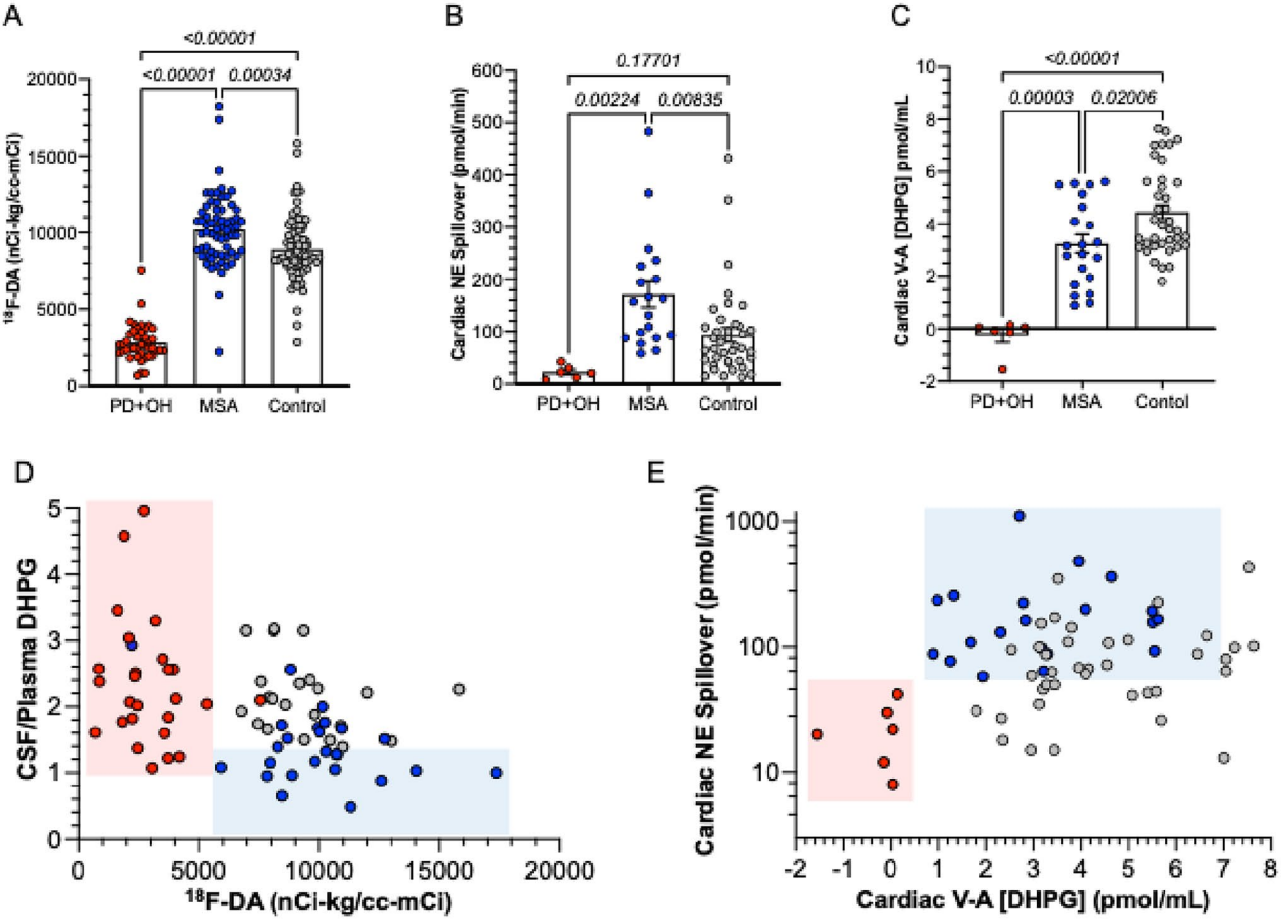
Biomarkers separating multiple system atrophy (MSA, blue) from Parkinson disease with orthostatic hypotension (PD+OH, red) and control subjects (gray). **A** Interventricular septal myocardial ^18^F-dopamine (^18^F-DA)-derived radioactivity; **B** cardiac norepinephrine (NE) spillover; **C** cardiac venous–arterial (V–A) difference in plasma 3,4-dihydroxyphenylglycol (DHPG); **D** CSF/plasma ratio of DHPG vs. cardiac ^18^F-DA-derived radioactivity; **E** cardiac NE spillover vs. cardiac venous–arterial (V–A) difference in plasma DHPG. In **D** and **E**, pink and blue rectangles are placed manually to emphasize differences between MSA and PD+OH. In **A** note decreased ^18^F-DA-derived radioactivity in PD+OH and increased radioactivity in MSA compared to controls, and in **B** low cardiac NE spillovers in PD+OH and elevated spillovers in MSA. Combining CSF/plasma ratios of DHPG with cardiac ^18^F-DA-derived radioactivity and combining cardiac NE spillovers with cardiac venous–arterial (V–A) difference in plasma DHPG efficiently separated MSA from PD+OH

Cardiac ^18^F-DA-derived radioactivity efficiently separated the MSA from the PD+OH groups (Fig. 3A), with the radioactivity decreased from control in PD+OH and increased from control in MSA. Similarly, cardiac NE spillovers were decreased from control in PD+OH and increased from control in MSA (Fig. 3B). Cardiac arteriovenous increments in plasma DHPG were strikingly decreased in PD+OH (Fig. 3C) and tended to be decreased in MSA. MSA and PD+OH were highly efficiently separated when individual values for CSF/plasma ratios of DHPG were expressed as a function of cardiac ^18^F-DA-derived radioactivity (blue and pink rectangles in Fig. 3D) and when cardiac arteriovenous increments in plasma DHPG were expressed as a function of cardiac NE spillover (blue and pink rectangles in Fig. 3E).

## Discussion

There is a longstanding need for pathophysiologically relevant biomarkers that can separate MSA from PD+OH. In the present retrospective analysis we obtained evidence that CSF/plasma ratios of DHPG, CSF/plasma ratios of NE, cardiac arteriovenous increments in plasma DHPG, cardiac NE spillovers, and cardiac ^18^F-DA-derived radioactivity all distinguish MSA from PD+OH. The following discussion explains why these findings make sense from a pathophysiological point of view.

CSF DHPG probably mainly reflects central neural stores of NE, which are depleted in both PD+OH and MSA [34]. In the periphery, however, there is sympathetic noradrenergic deficiency in PD+OH [37], whereas in MSA sympathetic noradrenergic innervation and function generally are intact [38, 39]. Accordingly, one would expect that CSF/plasma ratios of DHPG and NE would be lower in MSA than in PD+OH. When CSF/plasma ratios of DHPG and NE were considered together, none of the MSA patients had values for both variables within the control range, whereas most of the PD+OH patients did. These data suggest that the finding of low CSF/plasma ratios of DHPG and NE offers a positive biomarker for MSA compared to PD+OH. The relative preservation of peripheral DHPG levels in MSA despite central NE depletion likely reflects intact peripheral sympathetic innervation and normal intra-neuronal enzymatic metabolism of cytoplasmic NE, in contrast with denervation and a shift from vesicular sequestration to enzymatic metabolism in PD+OH [40, 41].

The sympathetic noradrenergic lesion in PD+OH is especially prominent in the heart [42]. Thus, we reported previously that patients with PD+OH have markedly decreased cardiac NE spillovers even when total body NE spillovers are within normal limits [33]. We have also reported that PD+OH entails attenuated cardiac arteriovenous increments in plasma DHPG levels [33]. In the present study we found that combining cardiac NE spillovers with cardiac arteriovenous increments in plasma DHPG levels completely separated MSA from PD+OH, whereas arm arteriovenous increments in plasma DHPG were ineffective in this regard.

The present neurochemical and sympathetic neuroimaging findings add to growing evidence from cross-sectional studies that one can separate groups of patients with MSA or PD+OH by biomarkers. Other biomarkers include measures of olfactory dysfunction [43], diffusion tensor magnetic resonance imaging [44], α-syn seeding activity [8], α-syn deposition in skin biopsies [9], and CSF neurofilament light chain [45]. The relative efficiencies of these and other modalities, alone and especially in combination, for distinguishing MSA from PD+OH remain to be assessed.

Overall, our data provide new support for DHPG measurements as a practical and potentially scalable clinical test for distinguishing MSA from PD+OH. Even without cardiac catheterization, the CSF/plasma DHPG ratio alone yields respectable separation between MSA and PD+OH in ROC analyses, suggesting potential utility in academic autonomic medicine centers.

### Limitations

The participants in this study were highly selected and studied at a single site, and they agreed to undergo comprehensive testing without the presumption of personal benefit. Generalizability to the overall population of autonomic synucleinopathy patients is unknown.

The research included invasive procedures such as right heart catheterization and lumbar puncture. In most of the patients there was no neuropathological confirmation of alpha-synucleinopathy.

Relatively few groups in the United States carry out right heart catheterizations and arterial blood sampling, and none measure cardiac NE spillover, which requires both infusion of tracer-labeled NE and means to quantify coronary sinus blood flow. The present data suggest that cardiac arteriovenous increments in plasma DHPG may be sufficient to separate MSA from PD+OH in individual patients. This seems within the capability of cardiology investigators who evaluate patients with neurocardiological disorders at academic centers [46–48].

Commercial laboratories do not currently offer DHPG assays, and liquid chromatography with electrochemical detection, which has been the standard method used for many years [35], is becoming impractical because of the lack of availability of electrodes suitable for series electrochemical detection. Perhaps the present results will help convince laboratories that assay catecholamines by liquid chromatography with tandem mass spectroscopy to add DHPG to their repertoires.

### Implications

This report introduces neurochemical biomarkers that separate PD+OH from MSA based on CSF and plasma DHPG levels. Prospective studies are needed to determine whether these biomarkers predict the trajectory of phenoconversion from clinically defined pure autonomic failure to central synucleinopathies [43, 49, 50]. Neurochemical biomarkers assessed in combination with other modalities such as skin biopsies with immunofluorescence confocal microscopy [51] or α-syn seed amplification assays [52] may lead to a multimodal framework that would enable efficient diagnosis by convergence of laboratory abnormalities.

## Data Availability

The Supplementary Data Workbook provides the data upon which the present manuscript is based.
